# *In vivo* neuronal firing patterns during human epileptiform discharges replicated by electrical stimulation

**DOI:** 10.1016/j.clinph.2012.02.062

**Published:** 2012-09

**Authors:** Gonzalo Alarcón, Juan Martinez, Shashivadan V. Kerai, Maria E. Lacruz, Rodrigo Quian Quiroga, Richard P. Selway, Mark P. Richardson, Jorge J. García Seoane, Antonio Valentín

**Affiliations:** a Department of Clinical Neuroscience, Institute of Psychiatry, King’s College London, United Kingdom; b Departamento de Fisiología, Facultad de Medicina, Universidad Complutense, Madrid, Spain; c Department of Engineering, University of Leicester, Leicester, United Kingdom; d Leibniz Institute for Neurobiology, University of Magdeburg, Germany; e Department of Neurosurgery, King’s College Hospital, London, United Kingdom

**Keywords:** IEDs, interictal epileptiform discharges, EEG, electroencephalogram, SPES, single pulse electrical stimulation, Extracellular recording, Single unit activity, Epileptiform discharge, Single pulse electrical stimulation, SPES, Epilepsy, Pathophysiology, Human, *In vivo*

## Abstract

**Objective:**

To describe neuronal firing patterns observed during human spontaneous interictal epileptiform discharges (IEDs) and responses to single pulse electrical stimulation (SPES).

**Methods:**

Activity of single neurons was recorded during IEDs and after SPES in 11 consecutive patients assessed with depth EEG electrodes and attached microelectrodes.

**Results:**

A total of 66 neurons were recorded during IEDs and 151 during SPES. We have found essentially similar patterns of neuronal firing during IEDs and after SPES, namely: (a) a burst of high frequency firing lasting less than 100 ms (in 39% and 25% of local neurons, respectively for IED and SPES); (b) a period of suppression in firing lasting around 100–1300 ms (in 19% and 14%, respectively); (c) a burst followed by suppression (in 10% and 12%, respectively); (d) no-change (in 32% and 50%, respectively).

**Conclusions:**

The similarities in neuronal firing patterns associated with IEDs and SPES suggest that, although both phenomena are initiated differently, they result in the activation of a common cortical mechanism, probably initiated by brief synchronised burst firing in some cells followed by long inhibition.

**Significance:**

The findings provide direct *in vivo* human evidence to further comprehend the pathophysiology of human focal epilepsy.

## Highlights

► The neuronal firing patterns during interictal epileptiform discharges (IEDs) and after single pulse electrical stimulation (SPES) can be described as burst-only, suppression-only, burst–suppression or no-change. ► Similar neuronal firing patterns can be observed during IEDs and after SPES. ► IEDs and responses to SPES appear to activate similar and generic cortical mechanisms, which may explain transient cognitive impairment.

## 1 Introduction

Epilepsy is one of the most common neurological conditions, affecting an estimated 0.5% of the population. Despite advances in neuroimaging, in EEG analysis, and extensive animal research over the last decades, many aspects of the pathophysiology of human focal epilepsy remain unclear. A typical feature of epilepsy is the spontaneous interictal epileptiform discharge (IED), which is seen using conventional clinical EEG recorded from scalp, as well as using intracranial EEG. Similar EEG phenomena can be induced in response to single pulse electrical stimulation (SPES) applied to the cortex in human epilepsy (Valentin et al., 2002, 2005a,b; Flanagan et al., 2009; Lacruz et al., 2007).

Understanding the mechanisms of human IEDs and responses to SPES would be of practical benefit. On the one hand, it is traditionally assumed that IEDs represent ‘mini seizures’ which are not long enough to become full seizures. Conversely, it has been suggested that IEDs are associated with a period of increased threshold to stimulation (Lebovitz, 1979; de Curtis and Avanzini, 2001) and may represent neuronal mechanisms protective of seizures. The clinical implications of the two interpretations of IEDs are distinctly different. Indeed, although the presence of IEDs is clearly associated with epilepsy, IEDs can occur in areas other than those originating clinical seizures (Alarcon et al., 1994, 1997).

Human *in vivo* EEG recordings and simultaneously recorded neuronal action potentials suggest a variable relation between IEDs and the activity of individual neurons (Babb et al., 1973; Keller et al., 2010; Wyler et al., 1982). Several experimental studies in tissue slices and in animal epilepsy models have concentrated on identifying the conditions associated with synchronous and excessive burst firing (Jiruska et al., 2010; Brown and Randall, 2009; Calvin et al., 1968; Jefferys, 1989, 2003). In animal and human epileptogenic cortex *in vivo*, the tendency for neurons to fire in bursts has been reported to be increased (Calvin et al., 1973; Babb and Crandall, 1976), decreased (Colder et al., 1996) or both (Goldenshon and Purpura, 1963).

There are very limited opportunities to study the behaviour of neurons in human subjects. Epilepsy patients suitable for surgical treatment are sometimes studied with intracerebral EEG electrodes (subdural electrodes or intracerebral depth electrodes) in order to record EEG activity from deep cortical structures and accurately identify the regions originating seizures. We have combined depth EEG electrodes with microelectrode recordings (Fried et al., 1999) in order to simultaneously record action potentials from individual neurons and the EEG generated by the surrounding neuronal network. During IEDs, human neurons *in vivo* have been reported to increase, decrease or not change their firing rates (Alarcon et al., 2008; Keller et al., 2010). In the present work, we report the duration, magnitude and spatial extent of the changes in neuronal firing seen during IEDs, and test the hypothesis that such changes can be induced by neuronal synchronization induced by SPES. We discuss the pathophysiological and clinical relevance of the findings.

## 2 Methods

### 2.1 Patients

The study includes 11 consecutive patients that fulfilled the following inclusion criteria: (a) assessed for epilepsy surgery with depth electrodes in King’s College Hospital, (b) gave written consent to have microelectrodes implanted, (c) showed action potentials in microelectrode recordings. All patients were fully informed of the nature of the research and gave informed consent according to the declaration of Helsinki. The experimental procedure was approved by the Local Research Ethics Committee of King’s College Hospital (reference number 02–003).

### 2.2 Electrodes

Patients were assessed with 6 contact depth EEG electrodes (AdTech Medical Instruments Corp., WI, USA), combined with six or seven 40 μm diameter platinum microelectrodes inserted through the hollow lumen of the electrode assembly (Fried et al., 1999). The electrodes were inserted in a lateral-to-medial direction, targeted at the amygdala, hippocampus or medial frontal cortex. Their placement was dictated only by clinical considerations and the experimental study had no bearing on these decisions.

### 2.3 Recordings

During recordings, patients were kept awake and were encouraged to relax in order to minimise movement artefacts. Single cell and multiunit extracellular activity recorded with the microelectrodes was analog bandpass filtered between 500 and 5000 Hz and digitised at 24 kHz with a 4-channel Leadpoint system (Medtronic Minneapolis, MN, USA). To reduce noise levels, neuronal activity was recorded as voltage difference between two microelectrodes from the same bundle.

Simultaneous EEG activity was recorded between two intracranial EEG electrodes in order to allow synchronization between IEDs and single unit activity. The EEG channel was bandpass filtered between 0.5 and 50 Hz.

### 2.4 Single pulse electrical stimulation (SPES)

Single pulse electrical stimulation was performed between adjacent macroelectrodes using a constant-current neurostimulator (Medelec ST10 Sensor, Oxford Instruments, United Kingdom). Electrical stimulation was carried out following the protocol described elsewhere (Valentin et al., 2002), using single monophasic pulses of 1 ms duration delivered every 10 s with a current intensity ranging between 6 and 8 mA.

### 2.5 Spike sorting and analysis

Data from microelectrode and simultaneous EEG recording were analysed and the activities of different neurons per microelectrode were identified by using Wave_clus as a spike sorting algorithm (Quiroga et al., 2004).

**Fig. 1:**
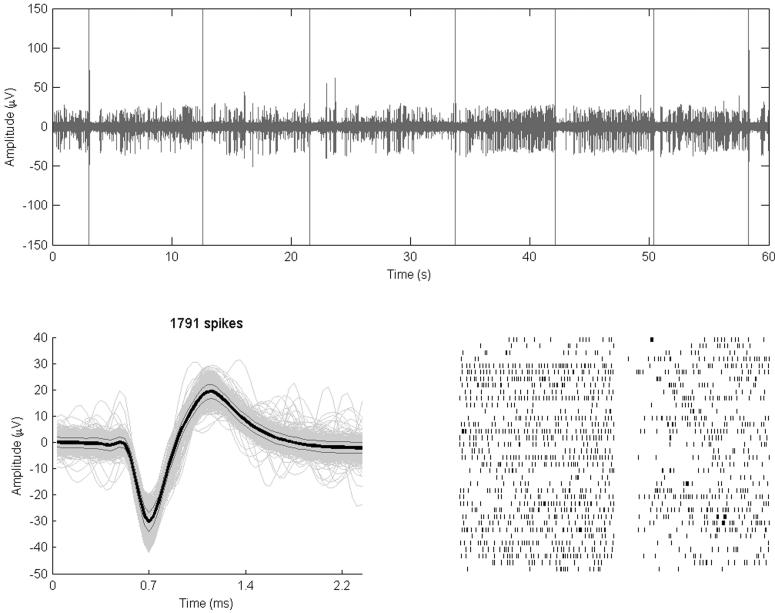
Example demonstrating how a peri-stimulation raster is obtained. The top trace represent 1 min of microelectrode recording. The long vertical lines show the stimulation artefact generated by a single electrical pulse occurring approximately every 10 s. Note that the recording contains action potentials of different amplitudes and polarities, generated by different neurons. Cells tend to go quiet for around 0.5 s after each stimulus. Action potentials of similar morphology, polarity and amplitude are identified by spike sorting software as coming from the same neuron. The left bottom graph shows all identified action potentials from one neuron superimposed on the average (thick trace) and ±1 SD. The right bottom graph shows the peri-stimulus raster plot for this neuron. Each row represents 8 s of recording centred at the stimulus (4 s before and 4 s after a pulse). Each dot represents the occurrence of a single action potential from the neuron. Successive stimulations are aligned to the stimulation artefact.

**Fig. 2:**
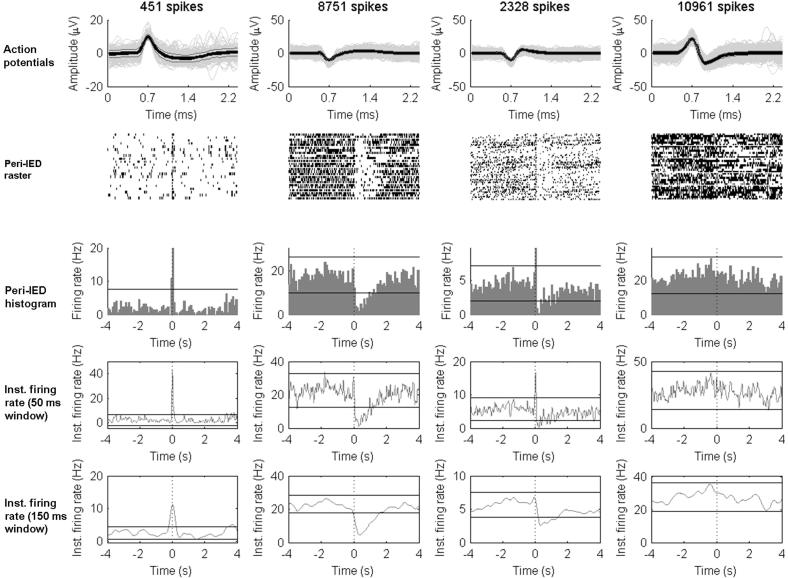
Examples of neuronal firing patterns during IEDs. For each column, the top graph shows the superposition of all action potentials recorded from the corresponding neuron. The second graph below shows the peri-IED raster plots of the cortical neuron 4 s before and 4 s after the synchronising peak of the simultaneously recorded IEDs on the EEG. Each row of the raster plot is aligned to the peak of the IED. Each dot represents the occurrence of a single action potential in the recorded neuron. The third graph below shows the peri-IED histogram with 100 ms bins during the same period. The fourth graph shows the instantaneous firing rate using a Gaussian window of 50 ms window during the same period. The parallel horizontal lines represent the mean ±3 SD of voltage during the baseline (the 4 s preceding the synchronising event). The bottom graph shows the instantaneous firing rate using a Gaussian window of 150 ms. The parallel horizontal lines represent the mean ±3 SD of voltage during the baseline. Note that for each figure the timescale of the top graph is different from all the others. From left to right, each column represents an example of a burst-only neuron, a suppression-only neuron, a burst–suppression neuron and a no-change neuron. Inst. = instantaneous.

**Fig. 3:**
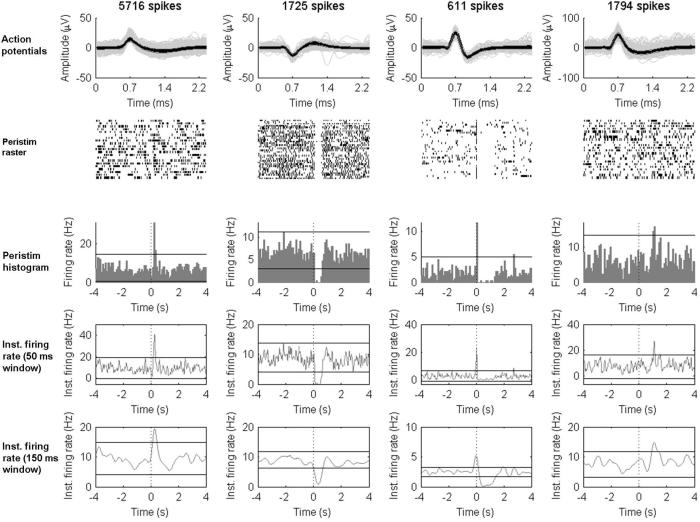
Examples of neuronal firing patterns during SPES. For each column, the top graph shows the superposition of all action potentials recorded from the corresponding neuron. The second graph below shows the peristimulus raster plots of the neuron 4 s before and 4 s after the stimulation pulse. Each dot represents the occurrence of a single action potential in the recorded neuron. The third graph below shows the peristimulus histogram with 100 ms bins during the same period. The fourth graph shows the instantaneous firing rate using a Gaussian window of 50 ms during the same period. The parallel horizontal lines represent the mean ±3 SD of voltage during the baseline (the 4 s preceding the stimulus). The bottom graph shows the instantaneous firing rate using a Gaussian window of 150 ms. The parallel horizontal lines represent the mean ±3 SD of voltage during the baseline. Note that for each figure the timescale of the top graph is different from all the others. From left to right, each column represents an example of a burst-only neuron, a suppression-only neuron, a burst–suppression neuron and a no-change neuron. Inst. = instantaneous; peristim = peri-stimulus.

Since neuronal firing rates show considerable temporal variability (Fig. 1, top trace), the time course of action potentials was shown as peri-event rasters (Fig. 1, right bottom graph) and peri-event histograms for each cluster (neuron). Microelectrode recordings from 4 s before and after each stimulus or IED were analysed (each of these 8 s epochs are called “trial”). Successive trials were synchronised with the sharpest peak of the IEDs or with the stimulation artefact for responses to SPES (Fig. 1, top trace). Only recordings containing more than five SPES pulses or IEDs were used for analysis. Peri-event rasters and histograms were initially visually inspected to identify firing patterns associated with SPES or IEDs. From this inspection four different patterns were identified: no-change, burst-only, suppression-only or burst–suppression. To quantify these patterns, the instantaneous firing rate was calculated as the convolution of the normalized spike trains by a Gaussian kernel (sampling period = 0.5 ms). To accommodate the different durations of patterns, two different Gaussian kernels with a standard deviation of 50 and 150 ms were used. For each window, upper and lower thresholds were calculated as the instantaneous firing rate ±3 standard deviations (SD) calculated during the 4 s preceding the synchronization events (spike peak for IEDs or stimulation artefact for SPES, Figs. 2 and 3).

A burst pattern was considered if the instantaneous firing rate crossed the upper threshold within 200 ms before or after the synchronising events. A suppression pattern was considered if the instantaneous firing rate crossed the lower threshold within 500 ms before or after the synchronising events. A burst–suppression pattern consisted of a burst pattern followed by a suppression pattern. The term burst–suppression refers to the behaviour of the firing rates of individual neurons, and is not related to the burst–suppression patterns seen on EEG recordings during sedation or during anoxic encephalopathy.

For a characterization of the burst and suppression responses, the following parameters were calculated:

*Baseline frequency:*The average of the instantaneous firing rate for the 4 s preceding the synchronising event.

*Amplitude of the suppression:*The lowest instantaneous firing rate during suppression.

*Duration of the suppression:*Difference in time since the instantaneous firing rate crossed downwards and upwards the −3 SD lower threshold below the baseline frequency.

*Latency of the suppression:*The latency of the lowest value of the instantaneous firing rate during suppression.

*Duration of the burst:*Difference in time since the instantaneous firing rate crossed upwards and downwards the +3 SD upper threshold above the baseline frequency.

*Amplitude of the burst:*Inverse of the maximum inter spike interval averaged across all the trials within the duration of the burst.

The following terms have been used to define the distance between the neurons and the areas recording IEDs or stimulated during SPES (IED/SPES areas):

*Local neurons:*Within 3 cm of the IED/SPES areas.

*Lobar neurons:*In the same lobe as the IED/SPES areas, but more than 3 cm away.

*Inter-lobar neurons:*In a different lobe but ipsilateral to the IED/SPES areas.

*Contralateral neurons:*In the contralateral hemisphere to the IED/SPES areas.

## 3 Results

### 3.1 Patients and neurons

Eleven patients were recruited into the study (4 males and 7 females). The mean age at the onset of epilepsy was 10.8 years (SD = 5.7). Two patients had history of febrile convulsions. The mean age at assessment was 35.2 years (SD = 13.2). Eight patients underwent surgery for the treatment of their epilepsy. Neuropathological examination showed mesial temporal sclerosis in four patients, nodular heterotopia in one patient, focal cortical dysplasia in two, and astrocytoma grade 2 plus mesial temporal sclerosis in one patient. After surgery, three patients enjoyed Engel outcome scale grade 1, two patients had grade 2, two patients had grade 3 and one patient had grade 4. Average follow-up was 28.9 months (SD = 18.4).

In 7 patients IEDs were recorded, and 10 patients underwent SPES simultaneous to microelectrode recordings. Two patients had only frontal electrodes, 7 patients had only temporal electrodes and 2 had frontal and temporal electrodes. Seizure onset was medial temporal in 7 patients, lateral temporal in one, lateral frontal in two and occipital in one.

During IED studies, 66 neurons were recorded with 34 microelectrodes (1.9 neurons per microelectrode). During SPES studies, 151 neurons were recorded with 87 microelectrodes (1.7 neurons per microelectrode). For IED rasters, a mean of 33.13 IEDs (SD = 28.44) were studied to calculate and display peri-IED raster plots, histograms and instantaneous firing rates. For SPES rasters, a mean of 21.5 stimuli (SD = 10.26) were applied to calculate and display peri-stimulus raster plots, histograms and instantaneous firing rates. Among the 66 neurons studied during IEDs, 13 were recorded at the seizure onset zone. Among the 151 neurons studied during SPES studies, 35 were recorded at the seizure onset zone and 116 neurons were recorded elsewhere.

### 3.2 Neuronal firing patterns

Four distinct patterns of neuronal firing rates were identified related to IEDs and SPES: burst-only, suppression-only, burst–suppression and no-change (Figs. 2 and 3).

### 3.3 Neuronal firing patterns during IEDs (7 patients, Fig. 2)

Only local and contralateral neurons were recorded during IEDs (Table 1). Thirty-two percent of local and 43% of contralateral neurons showed no-change in firing rate during IEDs. Among the neurons showing firing changes, burst-only was the most common pattern, followed by suppression-only and burst–suppression. Approximately half of local neurons showed an initial burst firing (either as burst-only or as burst–suppression) and nearly 30% showed longer lasting suppression (either as suppression-only or as burst–suppression). Neurons showing suppression (burst–suppression or suppression-only) showed higher baseline firing rates (mean = 7.97, SD = 8.18) than burst-only neurons or those showing no-change (mean = 2.84, SD = 3.85), and the difference was significant (Student *t*-test, two tailed, 57 *df*, *p* < 0.05). During bursts, the firing rate increased 3.5–22 times. During suppression, the firing rate decreased by 63.3% for suppression-only neurons and by 53.4% for burst–suppression neurons. Mean suppression durations were 2.8–6.3 times longer than mean burst durations (Table 1). The mean latency to the maximal suppression was 120 ms (burst–suppression neurons) or 153 ms (suppression-only neurons) after the synchronising peak of the IEDs. Among the 7 contralateral neurons, 4 were temporal and 3 were frontal, and none showed suppression.

Table 2 shows the characteristics of local temporal and frontal neurons. The proportion of neurons showing no-changes in the frontal lobe is higher than in the temporal lobe (64.3% versus 22.2%, two-tailed Fisher’s exact test, *p* = 0.007). Neurons showed behaviour largely following the features shown in Table 1 for the overall population. Temporal neurons showed higher baseline firing rates (mean = 5.3, SD = 6.4) than frontal neurons (mean = 1.2, SD = 1.54) and the difference is statistically significant (Student *t*-test, 57 *df*, *p* < 0.01). There were no frontal neurons showing burst patterns (neither burst-only, nor burst–suppression), and among suppression-only neurons, the amplitude of suppression was larger than for temporal neurons (a decrease in firing rate of 75.6% compared to baseline for frontal neurons versus 53.6% for temporal neurons, Student *t*-test, 15 *df*, *p* < 0.05).

### 3.4 Neuronal firing patterns during SPES (10 patients, Fig. 3)

Table 3 shows the characteristics of neurons presenting each firing pattern following SPES, according to distance to stimulation. The largest proportion of neurons showed no-changes in firing rate associated with stimulation, and this proportion increases the further location of the stimulus, ranging from 49.7% for local stimulation to 81.6% for contralateral stimulation.

As with IEDs, local neurons showing suppression (burst–suppression or suppression-only) after stimulation showed higher baseline firing rates (mean = 10.69, SD = 8.99) than burst-only neurons or those showing no-change (mean = 3.28, SD = 3.57), and the difference is statistically significant (Student *t*-test, 149 *df*, *p* < 0.01). During bursts after local stimulation, the mean of firing rates increased 5.2–9.3 times the baseline firing rate. The magnitude of the suppression for suppression-only and burst–suppression neurons is not statistically different. As during IEDs, the average durations of the suppression were much longer than those of the bursts (Table 3, local neurons): 506 ms (suppression-only) and 679 ms (bust-suppression) versus 73 ms (burst-only) and 97 ms (burst–suppression).

Table 4 shows the number and proportion of each firing pattern according to the lobe where the neuron was recorded. No inter-lobar frontal neurons were recorded. The number of neurons showing burst–suppression was minimal in the frontal lobe.

Table 5 shows the average and standard deviations for the baseline firing rates, amplitudes and durations of bursts and suppressions for local neurons in temporal and frontal locations. Frontal neurons showed no burst–suppression, and had shorter and earlier suppression than temporal neurons (Student *t*-test, 37 *df*, *p* < 0.01). Frontal neurons showed lower baseline firing rates (mean = 3.76, SD = 3.78) than temporal neurons (mean = 6.9, SD = 8.18), and the difference was statistically significant (Student *t*-test, 149 *df*, *p* < 0.01).

### 3.5 Proportions of neurons showing each response among IEDs and SPES

SPES showed a higher proportion of local neurons showing no-change than IEDs (49.7% versus 32.2%; Chi-square with Yates correction = 4.551, 1 *df*, two-tailed, *p* = 0.0329). Among the remaining local neurons, the percentage of neurons showing each response type (burst-only, suppression-only or burst–suppression) was similar among IEDs and SPES (57%, 28%, 15% versus 48%, 28%, 24%; Chi-square goodness of fit = 5.063, 2 *df*, *p* > 0.05).

### 3.6 Differences in firing rate parameters between IEDs and SPES responses

No differences were found in firing rate parameters of local neurons between IEDs and SPES (Tables 1 and 3, Student *t*-test at *p* < 0.05 plus Bonferroni correction for multiple comparisons). Differences tested were: baseline firing rate (150 ms window) in all four cell types, amplitude and duration of bursts for burst-only and burst–suppression neurons, and amplitude, duration and latency of suppression for suppression-only and burst–suppression neurons.

## 4 Discussion

### 4.1 Neuronal patterns

We have found essentially similar types of neuronal responses during IEDs and after SPES:

(a) Burst-only: A burst of high frequency firing generally lasting less than 100 ms (present in 39% and 25% of local neurons, respectively for IED and SPES).
(b) Suppression-only: A period of suppression in firing lasting around 100–1300 ms (present in 19% and 14%, respectively).
(c) Burst–suppression: A burst followed by suppression (present in 10% and 12%, respectively).
(d) No-change (present in 32% and 50%, respectively).

In addition to the description of these firing patterns and their incidence, we report the duration and magnitude of bursts and suppressions. Burst firing occurs before suppression, which is longer lasting. Moreover, if neurons showing no-change are excluded, the proportions of cells showing each response pattern (burst-only, suppression-only or burst–suppression) are similar for IEDs and SPES. The similarities in neuronal firing patterns associated with IEDs and SPES suggest that, although both phenomena are initiated differently, they result in the activation of a common cortical mechanism. This is further supported by the finding that such neuronal patterns can be seen in normal and abnormal areas in response to SPES.

A surprising finding is the long periods of suppression found, of up to 1.3 s, which may have clinical and pathophysiological implications as discussed below.

### 4.2 Relevance to the pathophysiology of human focal epilepsy

Our findings have the following implications relevant to the pathophysiology of human epilepsy:

(1) The finding that similar types of neuronal responses are observed during IEDs and after SPES, suggests that both phenomena activate the same neurophysiological mechanisms. The firing patterns observed start within 100 ms of stimulation, i.e. during early responses to SPES. Since early responses can be seen when stimulating at most cortical sites, regardless of their epileptogenic protential (Valentin et al., 2002), some events activated by IEDs and SPES may be part of a generic cortical mechanism rather than epilepsy-specific. This may explain why IEDs are often seen in areas other than those originating seizures (Alarcon et al., 1994, 1997).
(2) Between 30% and 50% of local neurons did not show clear changes in firing rate, suggesting that IED-like patterns can occur without the involvement of all nearby neurons, as reported previously for IEDs (Alarcon et al., 2008; Wyler et al., 1982; Fried et al., 1999).
(3) Since the patterns of neuronal firing described above can be induced by brief and localised electrical stimulation which simultaneously excites a proportion of local neurons, it would be reasonable to assume that IEDs are triggered by a sudden synchronization of interconnected neurons showing burst or burst–suppression. Furthermore, these cells are only around 36% of local neurons, suggesting that the majority of neurons need not be synchronized in order to result in IEDs.
(4) Immediately preceding IEDs, around 12% of neurons significantly increase and 7.6% significantly decrease their firing rate (Keller et al., 2010). Our finding that IED-like events can be generated by SPES, necessarily without related preceding changes in firing rate, suggests that such changes observed before IEDs are not an intrinsic part of IEDs. They may represent the phenomena responsible for setting the conditions for IEDs to occur, or may result from random fluctuations in the baseline firing rates.
(5) The magnitude and timescale of the neuronal firing changes are remarkable. Whereas the initial burst firing is rather brief, lasting for less than 100 ms, the suppression is much longer, lasting for up to 1300 ms. The initial burst firing could be responsible for ripple and fast-ripple activity that can be recorded following SPES (van ‘t Klooster et al., 2011). The long duration for suppression is unlikely to result solely from paroxysmal depolarizing shifts or from intrinsic membrane properties. Furthermore, the finding that some cells showed ‘suppression-only’, without previous firing, would suggest that the suppression does not result from the intrinsic properties of the membrane but from the properties of the neuronal network, namely, that suppression is due to lateral or recurrent inhibition, or to a subcortical loop. We hypothesize that the neuronal firing changes observed are due to burst firing followed by a wave of recurrent inhibition responsible for the suppression. Indeed, extracellular stimulation of the human cortex can induce excitatory postsynaptic potentials followed by inhibitory postsynaptic potentials, presumably via recurrent inhibition (Schwartzkroin et al., 1983; Avoli and Olivier, 1989; McCormick, 1989).
(6) The presence of significant suppression during IEDs shows that IEDs can occur in cortical regions maintaining substantial inhibitory function, although inhibition may be altered. Furthermore, in slices from human patients with epilepsy, spontaneous hyperpolarizing inhibitory postsynaptic potentials were detected in 27% of CA2 neurons (Wittner et al., 2009), a percentage similar to the proportion of neurons showing suppression in the present study.
(7) The functional consequence of the long suppression periods is uncertain. Whereas in some regions, such long periods of inhibition may protect from seizures, in others they may be the cause of rebound synchronization as a significant number of cells may start firing synchronously shortly after inhibition ceases (Pavlov and Kullmann, 2010; Isokawa-Akesson et al., 1989). In particular, the existence of burst–suppression patterns imply that the same neuron can undergo suppression following burst firing, which might represent a protective mechanism against generalization.

### 4.3 Clinical implications

The long periods of neuronal inhibition associated with IEDs have clinical implications. Normal neuronal function in the involved regions may be compromised during the suppression period, as around nearly 30% of cells can remain silent during hundreds of milliseconds. This may be the neurophysiological mechanism underlying transient cognitive impairment described during focal IEDs in humans (Aarts et al., 1984) and memory impairment induced by hippocampal stimulation with a single electrical pulse (Lacruz et al., 2010).

### 4.4 Contralateral activation

It is surprising that some cells are activated by contralateral stimulation and during IEDs restricted to contralateral cortex. The proportion of cells showing no-change was higher among contralateral than among local neurons, and the majority of contralateral neurons were burst-only. This suggest that contralateral events can activate burst-only cells but are not strong enough to trigger a wave of inhibition, again suggesting that the initial event is the activation of burst cells.

## 5 Conclusion

Our results suggest that IEDs and SPES trigger similar normal neurophysiological mechanisms probably initiated by brief synchronised burst firing in some cells followed by long inhibition. These findings provide direct *in vivo* human evidence to comprehend our current models of focal epilepsy during the interictal period.

## Figures and Tables

**Table 1:** Firing patterns of local and contralateral neurons to IEDs.

	Number of cells (%)	Baseline firing rate in Hz	Burst Amplit in Hz	Burst Dur in ms	Suppress Amplit in Hz	Suppress Dur in ms	Suppress Lat in ms
		Mean (SD)	Mean (SD)	Mean (SD)	Mean (SD)	Mean (SD)	Mean (SD)
*Local*							
No-change	19 (32.2)	2.4 (4.1)	NA	NA	NA	NA	NA
Burst-only	23 (39)	3.1 (3.7)	68.2 (112.8)	94.6 (51.6)	NA	NA	NA
Suppression-only	11 (18.6)	6.0 (6.7)	NA	NA	2.2 (1.8)	539 (488)	153 (181)
Burst–suppression	6 (10.2)	11.6 (10.0)	40.0 (38.8)	85.2 (35.7)	5.4 (4.9)	269 (350)	120 (292)

*Contralateral*							
No-change	3 (42.9)	0.5 (0.4)	NA	NA	NA	NA	NA
Burst-only	4 (57.1)	4.9 (6.6)	17.65 (32.2)	61.4 (54.7)	NA	NA	NA
Suppression-only	0	NR	NR	NR	NR	NR	NR
Burst–suppression	0	NR	NR	NR	NR	NR	NR

Amplit = amplitude; Dur = duration; Suppress = suppression; ms = milliseconds; Lat = latency; SD = standard deviation; NA = not applicable; NR = none recorded. No lobar or interlobar neurons were recorded.

**Table 2:** Firing patterns in local neurons during IEDs according to lobe.

	Number of cells (%)	Baseline firing rate in Hz	Burst Amplit in Hz	Burst Dur in ms	Suppress Amplit in Hz	Suppress Dur in ms	Suppress Lat in ms
		Mean (SD)	Mean (SD)	Mean (SD)	Mean (SD)	Mean (SD)	Mean (SD)
*Temporal*							
No-change	10 (22.2)	4.1 (5.2)	NA	NA	NA	NA	NA
Burst-only	23 (51.1)	3.1 (3.7)	68.2 (112.8)	94. (51.6)	NA	NA	NA
Suppression-only	6 (13.3)	9.3 (7.5)	NA	NA	3.5 (1.3)	707 (614)	230 (219)
Burst–suppression	6 (13.3)	11.5 (10.0)	39.9 (38.8)	85.2 (35.7)	5.4 (4.9)	269 (350)	120 (292)

*Frontal*							
No-change	9 (64.3)	0.7 (0.5)	NA	NA	NA	NA	NA
Burst-only	0	NR	NR	NR	NR	NR	NR
Suppression-only	5 (35.7)	2.1 (2.4)	NA	NA	0.59 (0.7)	337 (180)	60 (40)
Burst–suppression	0	NR	NR	NR	NR	NR	NR

Amplit = amplitude; Dur = duration; Suppress = suppression; ms = milliseconds; Lat = latency; SD = standard deviation; NA = not applicable; NR = none recorded. No lobar or interlobar recordings were obtained.

**Table 3:** Firing patterns of local, lobar, interlobar and contralateral neurons after SPES.

	Number neurons (%)	Baseline firing rate in Hz	Burst Amplit in Hz	Burst Dur in ms	Suppress Amplit in Hz	Suppress Dur in ms	Suppress latency in ms
		Mean (SD)	Mean (SD)	Mean (SD)	Mean (SD)	Mean (SD)	Mean (SD)
*Local*							
No-change	75 (49.7)	3.5 (3.7)	NA	NA	NA	NA	NA
Burst-only	37 (24.5)	2.9 (3.2)	18.1 (22.5)	72.8 (38.6)	NA	NA	NA
Suppression-only	21 (13.9)	12.8 (10.4)	NA	NA	5.3 (7.2)	506 (458)	280 (144)
Burst–suppression	18 (11.9)	8.2 (6.4)	62.9 (90.0)	96.6 (55.5)	2.1 (1.8)	679 (447)	487 (140)

*Lobar*							
No-change	86 (72.9)	3.5 (4.0)	NA	NA	NA	NA	NA
Burst-only	24 (20.3)	2.3 (2.7)	7.1 (9.2)	47.8 (25.5)	NA	NA	NA
Suppression-only	7 (5.9)	9.1 (3.4)	NA	NA	2.8 (1.9)	494 (572)	153 (55)
Burst–suppression	1 (0.8)	5.5	4.7	55.0	0.37	57	109

*Interlobar*							
No-change	16 (72.7)	15.5 (13.3)	NA	NA	NA	NA	NA
Burst-only	4 (18.2)	8.9 (8.5)	19.8 (15.0)	72.8 (41.2)	NA	NA	NA
Suppression-only	2 (9.1)	14.2 (11.0)	NA	NA	5.7 (5.4)	34.5 (1.4)	156 (207)
Burst–suppression	0	NR	NR	NR	NR	NR	NR

*Contralateral*							
No-change	120 (81.6)	5.7 (6.8)	NA	NA	NA	NA	NA
Burst-only	22 (15)	2.0 (2.2)	8.6 (18.0)	38.5 (20.4)	NA	NA	NA
Suppression-only	4 (2.7)	7.4 (2.3)	NA	NA	3.8 (1.9)	216 (215)	8.1 (243)
Burst–suppression	1 (0.7)	7	18.1	51	3.8	226	−323

Amplit = amplitude; Dur = duration; Suppress = suppression; ms = milliseconds; NA = not applicable; NR = none recorded.

**Table 4:** Neurons showing each firing pattern according to lobe and distance to SPES.

	Local		Lobar		Inter-lobar		Contralateral	
	Number	%	Number	%	Number	%	Number	%
*Temporal*								
No-change	18	26.1	19	70.4	16	72.7	26	72.2
Burst-only	21	30.4	8	29.6	4	18.2	6	16.7
Suppression-only	12	17.4	0	0	2	9.1	4	11.1
Burst–suppression	18	26.1	0	0	0	0	0	0
Total	69	100	27	100	22	100	36	100

*Frontal*								
No-change	57	69.5	67	73.6	NR	NR	94	84.7
Burst-only	16	19.5	16	17.6	NR	NR	16	14.4
Suppression-only	9	11.0	7	7.7	NR	NR	0	0
Burst–suppression	0	0	1	1.1	NR	NR	1	0.9
Total	82	100	91	100	NR	NR	111	100

NR = none recorded.

**Table 5:** Firing patterns of local neurons following SPES according to lobe-Mean (standard deviation).

	Baseline firing rate (Hz)	Burst Amplit (Hz)	Burst Dur (ms)	Suppress Amplit (Hz)	Suppress Dur (ms)	Suppress latency (ms)
*Temporal*						
No-change	4.6 (4.6)	NA	NA	NA	NA	NA
Burst-only	2.3 (2.0)	20.3 (25.8)	77.4 (35.5)	NA	NA	NA
Suppression-only	16.5 (12.5)	NA	NA	8.1 (8.5)	691 (500)	333 (146)
Burst–suppression	8.2 (6.4)	62.9 (90.0)	96.6 (55.5)	2.2 (1.8)	679 (447)	487 (140)

*Frontal*						
No-change	3.1 (3.4)	NA	NA	NA	NA	NA
Burst-only	3.7 (4.2)	15.1 (17.7)	66.7 (42.8)	NA	NA	NA
Suppression-only	7.8 (2.9)	NA	NA	1.5 (1.5)	258 (243)	211 (115)
Burst–suppression	NR	NR	NR	NR	NR	NR

Amplit = amplitude; Dur = duration; Suppress = suppression; ms = milliseconds; NA = not applicable; NR = none recorded.
